# Very-Low-Calorie Ketogenic Diet: A Potential Treatment for Binge Eating and Food Addiction Symptoms in Women. A Pilot Study

**DOI:** 10.3390/ijerph182312802

**Published:** 2021-12-04

**Authors:** Elvira Rostanzo, Marco Marchetti, Ilenia Casini, Anna Maria Aloisi

**Affiliations:** 1 Department of Medicine, Surgery and Neuroscience, University of Siena, 53100 Siena, Italy; elvirarostanzo@gmail.com (E.R.); ilenia.casini@student.unisi.it (I.C.); 2 PhD School of Applied Medical-Surgical Sciences, University of Rome “Tor Vergata”, 00133 Rome, Italy; marco@marcomarchetti.it

**Keywords:** VLCKD, ketogenic diet, eating disorders, food addiction, binge eating, YFAS, behavioral addiction

## Abstract

Background: many patients who struggle to lose weight are unable to cut down certain ultra-processed, refined types of food with a high glycemic index. This condition is linked to responses similar to addiction that lead to overeating. A very-low-calorie ketogenic diet (VLCKD) with adequate protein intake could be considered a valid dietary approach. The aim of the present study was to evaluate the feasibility of a VLCKD in women with binge eating and/or food addiction symptoms. Methods: subjects diagnosed with binge eating and/or food addiction symptoms (measured with the Binge Eating Scale and the Yale Food Addiction Scale 2.0) were asked to follow a VLCKD with protein replacement for 5–7 weeks (T1) and a low-calorie diet for 11–21 weeks (T2). Self-reported food addiction and binge eating symptoms and body composition were tested at T0 (baseline) and at the end of each diet (T1 and T2 respectively); Results: five women were included in the study. Mean age was 36.4 years (SEM = 4.95) and mean BMI was 31.16 (SEM = 0.91). At T0, two cases of severe food addiction, one case of mild food addiction, one case of binge eating with severe food addiction, and one case of binge eating were recorded. Weight loss was recorded at both T1 and T2 (ranging from 4.8% to 11.6% of the initial body weight at T1 and from 7.3% to 12.8% at T2). No case of food addiction and/or binge eating symptoms was recorded at T2. Muscle mass was preserved. Conclusions: recent findings have highlighted the potential therapeutic role of ketogenic diets for the treatment of addiction to high-calorie, ultra-processed and high-glycemic food. Our pilot study demonstrates the feasibility of a ketogenic diet in women with addictive-like eating disorders seeking to lose weight.

## 1. Introduction

Individuals with overweight or obesity and who suffer from an eating disorder often report the desire to lose weight and improve their health. At the same time, however, they are unable to cut down certain types of foods, showing food addiction symptoms and lack of control.

According to the Diagnostic and Statistical Manual of Mental Disorders, Fifth Edition (DSM-5), the key diagnostic features of binge eating disorder (BED) include recurrent and persistent episodes of binge eating, marked distress regarding binge eating, absence of regular compensatory behaviors, and binge eating episodes associated with a variety of physical and psychological symptoms [1]. Moreover, food addiction is described as an addictive-like response to high sugar and high caloric consumption of food: food addiction symptoms include much time spent obtaining food, feelings of withdrawal when off food, continued use despite knowledge of adverse consequences, important activities reduced or given up, repeated unsuccessful attempts to quit, and food taken in larger quantities or for longer periods than intended [2].

The most widely used tool to evaluate food addiction is the Yale Food Addiction Scale [3] developed in 2009 by modeling all of the DSM-IV criteria for substance dependence for application to eating behavior. Later, the DSM-5 introduced significant changes to the substance-related and addictive disorders section, and a new version of the scale was developed in line with the DSM-5, called the Yale Food Addiction Scale 2.0 [4]. A positive Yale Food Addiction Scale diagnosis is usually positively associated with body mass index (BMI) and strongly linked with the presence of binge eating, although certain exceptions have been reported in the literature [5].

A recent study in a non-clinical sample of the Italian population estimated the prevalence of food addiction at 15.5%, and 84.2% of food-addicted subjects were classified as overweight or obese [6].

One of the most important goals for clinicians is to find an effective long-term dietary approach for people with food addiction and binge eating symptoms who wish to lose weight, i.e., an approach that avoids weight regain and relapses.

Individuals with BED appear to be at greater risk than those without the disorder of difficulties during and after a very-low-calorie diet program. Subjects with BED had a greater likelihood of greater lapses in adherence during the modified fast and refeeding, early major regain of lost weight, and poor outcome at one year [7].

While there is a lack of knowledge about the effects of a ketogenic diet on binge eating symptoms, the treatment of individuals with BED known to have long histories of failed attempts at dieting by means of a strict ketogenic diet may be associated with a rebound of binging. Hence, for successful weight loss, it is fundamental to propose both a very-low-calorie ketogenic diet (VLCKD) and behavioral therapy.

The carbohydrate-insulin model of obesity has shown that the consumption of processed, high-glycemic load carbohydrates produces hormonal changes that promote calorie deposition in adipose tissue, exacerbates hunger, and lowers energy expenditure [8]. This suggests that a combination of calorie restriction and a diet low in refined and processed sugar could be a valid dietary approach.

Ketogenic diets include dietary treatments characterized by a reduction in carbohydrates (usually less than 50 g/die) and a relative percentage increase in fat and protein [9]. Ketosis is a physiological process in which the body’s principal energy source is fat. Physiological ketosis is different from the pathological ketoacidosis seen in type 1 diabetes: in diet-induced ketosis, ketonemia reaches maximum levels of 7/8 mmol/l and with no change in pH, whereas in uncontrolled diabetic ketoacidosis it can exceed 20 mmol/l with a concomitant lowering of blood pH [10].

Moreover, one of the clinical benefits of a ketogenic diet is the prevention of an increase in appetite, despite weight loss; indeed, individuals may feel slightly less hungry (or fuller or more satisfied) [11]. The mechanism of appetite suppression is not established, although evidence supports a direct action of ketone bodies together with modifications in the levels of hormones that influence appetite, such as ghrelin and leptin [12].

Recently, a case report described remission from chronic anorexia nervosa by means of a ketogenic diet and ketamine [13], resulting in a surprising and long-lasting complete remission which has continued despite the stresses of living in New York City in the midst of the COVID-19 pandemic.

A recent case series [14] demonstrated the feasibility of a ketogenic diet for the treatment of food addiction and binge eating symptoms.

The aim of the present pilot study was to evaluate the effects of a very-low-calorie ketogenic diet followed by a low-calorie diet on self-reported food addiction and/or binge eating symptoms, its efficacy for weight loss, and its effects on body composition.

## 2. Materials and Methods

Patients presenting food addiction and/or binge eating symptoms were asked to follow a VLCKD with protein replacement for a period of 5–7 weeks and then to follow a low-calorie diet for a further 11–21 weeks. Self-reported food addiction and binge eating symptoms and body composition were tested at baseline (T0) and the end of each diet (T1 and T2, respectively). Food addiction was assessed using the Italian version of the Yale Food Addiction Scale 2.0 (I-YFAS 2.0) [15], while the Binge Eating Scale (BES) [16] was administered to evaluate binge eating symptoms.

The I-YFAS 2.0 is a self-reported questionnaire to assess addiction-like eating behavior during the past 12 months. The scale consists of 35 items scored on an eight-point scale ranging from never (score = 0) to every day (score = 7). The YFAS 2.0 symptom count is calculated as the sum of the number of fulfilling diagnostic criteria (ranging from 0 to 11). Food addiction diagnosis also requires the presence of impairment\distress criteria. The severity level of food addiction is calculated as:

Mild FA (with 2–3 symptoms and impairment\distress criteria);

Moderate FA (with 4–5 symptoms and impairment\distress criteria);

Severe FA (with ≥ 6 symptoms and impairment\distress criteria).

The Binge Eating Scale is a 16-item questionnaire constructed to describe both behavioral manifestations (e.g., eating large amounts of food) and feelings/cognitions surrounding a binge episode (e.g., guilt, fear of being unable to stop eating) [16]. A BES score ≥17 is an indication of binge eating symptoms, although there is no research to show that it can validly diagnose cases of binge eating disorder such as the DSM-5.

Anthropometric measurements were taken. The bodyweight of the patients, in underwear and without shoes, was measured to the nearest 0.1 kg on an electronic device (SECA^®^). Height was calculated with a stadiometer (SECA^®^) to the nearest 0.1 cm and BMI was calculated as weight (kg)/height (m^2^). Body circumferences were determined with a measuring tape and were used to evaluate body composition (fat mass and fat-free mass) using the equations of De Lorenzo et al. [17] to estimate fat mass in an Italian population.

The body composition results were taken into account to prescribe a suitable ketogenic diet with amino acid replacement. All the patients followed a VLCKD with an energy intake of around 1000 kcal/die (≤ 25 g/die of carbohydrates). The daily protein intake was calculated according to fat free mass, and 60% of the total protein intake was achieved using an amino acid supplement made of isolated whey protein (Macresces, Italfarmacia, Rome).

The patients followed a VLCKD for 5–7 weeks, according to the patient’s compliance, after which the same questionnaires were administered and the same instruments were applied under the same conditions to test for any changes (T1). All the patients tolerated the diet and reported no adverse effects. Diet adherence was tested through urinary ketone excretion, as measured by keto-sticks, until the end of the VLCKD.

After the VLCKD, and without an interval period, the patients followed a low-calorie diet, after which they were tested again with the same instruments and under the same conditions (T2). Only one patient did not fill out the questionnaires at this point.

The selection of these patients by the authors was not based on the amount of weight or fat mass lost but on the specific initial presentation of binge eating and/or food addiction symptoms. The patients were taking part in a study on mental well-being and nutrition approved by the Ethics Committee for Research in the Human and Social Sciences (CAREUS) of the University of Siena with protocol number 2742. Among the first 50 patients, it was suggested to those with a diagnosis of food addiction and/or binge eating symptoms and with the desire to lose weight and improve their body composition to adopt a very-low-ketogenic diet followed by a low-calorie diet. Selected patients were not taking medications or had other comorbidities. The study was conducted in full agreement with national and international regulations and the Declaration of Helsinki (2000). Limitations of the selection of participants should be noted. Only participants with the desire to lose weight among those with food addiction and/or binge eating symptoms were enrolled.

### Statistical Analysis

Comparisons of data for each variable (weight, BMI, fat mass, fat-free mass, waist-hip ratio) were carried out by analysis of variance (ANOVA)with the factor test (three levels: T0, T1, and T2 repeated), after a normality check (Kolmogorov–Smirnov and Lilliefors test) and sphericity test (Mauchly test). Post hoc analysis was carried out by Fisher’s LSD test. A level of *p* ≤ 0.05 was considered statistically significant. All analyses were performed with Statistica^®^ software (StatSoft Inc, Tulsa, United States). Data are presented as the mean ± standard error of the mean (SEM).

## 3. Results

Five women completed all the procedures. The mean age was 36.4 years old (SEM = 4.95), mean weight was 83 kg (SEM = 1.51) and mean BMI was 31.16 (SEM = 0.91) at T0.

At baseline (T0), two of the five patients reported a severe food addiction diagnosis (Table 1—#1 and #5), one reported mild food addiction diagnosis (#3), one reported binge eating symptoms with severe food addiction diagnosis (#4) and one reported only binge eating symptoms (#2).

VLCKD was found to be a feasible treatment since already at T1 all the patients reported improvements in the food addiction and/or BES score, as shown in Table 1. At T1, only one patient reported mild food addiction, and no binge eating symptoms were noted, according to the BES questionnaire. At T2, no case of food addiction or binge eating was reported. Patient #2 outcomes are unavailable because the participant was unable to go to the scheduled appointment to take measurements and complete the questionnaires. The participant was contacted by phone and reported that she was feeling improvements regarding binge eating symptoms and was continuing to follow the prescribed diet with no weight regain.

As shown in Table 2, at T1, the patients’ weight loss ranged from 4.8% to 11.6% of the initial body weight. ANOVA applied to bodyweight revealed a significant effect of the factor test (F(2,6) = 41.11, *p* < 0.001) due to T1 and T2 being significantly lower than T0 (*p* < 0.001 both). There was no significance between the weight measured at T1 and at T2.

The decrease in BMI ranged from 1.4 to 3.9. ANOVA applied to BMI revealed a significant effect of the factor Test (F (2,6) = 26.84, *p* = 0.001) due to T1 and T2 being lower than T0 (*p* < 0.001 both). There was no significance between the BMI measured at T1 and at T2. 

ANOVA applied to waist-hip ratio revealed a significant effect of the factor Test (F (2,6) = 6.41, *p* < 0.05) due to T2 being lower than T0 (*p* = 0.01). There was no significance between the waist-hip ratio measured at T0 and T1 and at T1 and T2.

ANOVA applied to fat mass revealed a significant effect of the factor Test (F (2,6) = 31.37, *p* < 0.001) due to T1 and T2 being lower than T0 (*p* < 0.001 both). There was no statistical significance between the fat mass at T1 and T2.

ANOVA applied to fat-free mass did not show any significant difference. Thus, the weight loss was only at the expense of fat mass, as fat-free mass was preserved.

## 4. Discussion

### 4.1. Ketogenic Diet and Food Addiction

The main result of the study was the reduction in symptoms of binge eating episodes and food addiction as measured by the YFAS 2.0 and BES questionnaires. All the patients showed, in addition to an improvement of food addiction and/or binge eating symptoms already at T1, weight loss and fat mass loss. Moreover, at T2, no patients showed a positive diagnosis of food addiction and/or binge eating symptoms.

McClernon et al. [18] examined the effect of diet on mood, hunger, and other associated symptoms in obese patients. They found hunger reduction and lower food cravings and a reduced negative effect in ketogenic diet-fed patients compared with those on a low-fat diet.

Food addiction and binge eating symptoms are associated with the consumption of high-glycemic foods rich in sugars, which are excluded in a VLCKD. Sethi Dalai et al. [19] supported the use of a low-carbohydrate ketogenic diet as a therapeutic approach for ultra-processed food addiction and binge eating symptoms.

Ketogenic diets have been shown to prevent an increase in ghrelin secretion, otherwise seen with weight loss, as well as to reduce and/or prevent hunger. Although the exact threshold of ketosis needed to induce appetite suppression and the mechanisms that mediate such an effect have yet to be elucidated [20], such diets could be a useful strategy to prevent craving for food in patients with binge eating. Moreover, the stabilization of blood glucose levels due to the ketogenic diet could reduce food craving and improve energy levels [21].

The therapeutic potential of ketogenic diets in several diseases has been reported in the literature, the most validated being obesity and refractory epilepsy. They have an emerging role in the treatment of neurological disorders, cancer, NAFLD, type 2 diabetes, and chronic pain among many others [22], but less is known about their use to treat binge eating and addiction to ultra-processed food.

Ketogenic diets have proved to be a useful strategy also in other behavioral addictions. Studies demonstrated that a ketogenic diet decreases alcohol intake in adult male mice [23] and diminishes behavioral responses to cocaine in young adult male and female rats [24]. Therefore, more studies assessing the effect of different ketogenic diets on addiction in humans are needed. The explanation of the potential benefit could be heterodimerization of the D1/D5 and D2/D3/D4 dopamine receptor families, which couple positively and negatively, respectively, to adenylyl cyclase. Heterodimers are formed by: D1 and adenosine A1 receptors, D2 or D3 and adenosine A2 receptors, and D2 and somatostatin SST5 receptors [25].

Although activation of the A1 receptor has been demonstrated to be one of the main anti-seizure mechanisms of the ketogenic diet [26], it seems that there is more evidence regarding the A2 receptor in the addiction field. Hence, researchers have proposed the adenosine-dopamine binomial as an interesting target that deserves to be explored in mental and substance-use disorders [23].

### 4.2. Ketogenic Diet and Body Composition

All the patients tolerated the VLCKD and reported weight loss after both the VLCKD and the low-calorie diet.

Interestingly, a recent meta-analysis showed that the use of whey protein supplementation may reduce the long and short-term appetite and induce a significant reduction in prospective food consumption [27]. The main effect of supplementation with whey protein in humans with obesity is a decrease in body fat mass and an improvement of metabolic syndrome parameters. Coupled with calorie restriction and exercise, these favorable effects persist while fat-free mass is maintained [28].

Importantly we showed that a VLCKD induced fat mass loss without a decrease of fat-free mass. Assessment of the participants’ body composition in order to prescribe a personalized VLCKD protocol is fundamental to evaluate the correct daily protein intake and prevent muscle mass loss.

A VLCKD with protein supplementation proved to be highly effective in terms of bodyweight reduction without inducing lean body mass loss compared with a very-low restricted-calorie diet [29].

The limitations of this study should be noted. A larger sample with control conditions is required. Further studies will be needed to assess the general acceptability of this diet among patients and its long-term effects on food addiction and binge eating symptoms. One of the main limitations in prescribing a ketogenic diet is the difficulty to maintain adherence over time, and the long-term effects of a ketotic state on metabolic health, such as basic blood and liver parameters, need to be better elucidated. Moreover, the order of interventions should be noted as a potential limitation on diet outcomes.

## 5. Conclusions

Our study strongly suggests the feasibility of a VLCKD in the treatment of a group of women with self-reported binge eating and food addiction symptoms. After a maintenance low-calorie diet, the patients experienced a reduction of food addiction and/or binge eating symptoms.

The patients showed a weight loss associated only with fat mass, while their muscle mass was preserved and their metabolic health improved.

Having a feasible nutritional approach to limit overeating and food addiction symptoms may help healthcare professionals find a reliable treatment to favor weight loss. In this view, a ketogenic diet may be a novel therapy for people with addictive-like eating disorders, helping to lower the sensation of hunger and craving.

This pilot study described the possibility of assessing a ketogenic protocol in patients with food addiction and\or binge eating, in the absence of other pathologies, where other interventions have failed. Future research to explore the role of nutritional ketosis in addiction symptoms in humans is needed.

## Figures and Tables

**Table 1:** Results of food addiction (FA) and binge eating scale (BES) questionnaires in the 5 subjects. Food addiction diagnosis is reported in 4 levels (no, mild, moderate, severe). Weeks refer to the time of observation.

Case	FA Diagnosis	FA Symptoms Count	BES Score	Weeks	FA Diagnosis	FA Symptoms Count	BES Score	Weeks	FA Diagnosis	FA Symptoms Count	BES Score
	**T0**			**T1**				**T2**			
**#1**	severe	7	6	6	no	0	4	11	no	0	3
**#2**	no	1	18	7	no	0	10		nd	nd	nd
**#3**	mild	3	15	7	no	2	11	15	no	0	9
**#4**	severe	10	29	5	mild	3	6	21	no	1	6
**#5**	severe	9	15	6	no	1	10	12	no	1	11
**Mean ± SEM**		6 ± 1.73	16.6 ± 3.70			1.2 ± 0.58	8.2 ± 1.36			0.5 ± 0.29	7.25 ± 2.02

Nd = not detected. T0 = baseline; T1 = 5–7 weeks; T2 = 11–21 weeks.

**Table 2:** Body composition outcomes recorded at baseline (T0), after 5–7 weeks (T1) and after 11–21 weeks (T2).

	T0	T1	T2
Weight (kg)	83 ± 1.52	76.2 ± 1.01 ***	75.75 ± 0.85 ***
BMI	31.16 ± 0.91	28.56 ± 0.63 ***	27.7 ± 0.58 ***
Fat mass (kg)	36.43 ± 1.28	31.04 ± 1.03 ***	29.23 ± 1.16 ***
Fat free mass (kg)	46.57 ± 1.09	45.16 ± 1.17	46.51 ± 0.65
Waist/Hip Ratio	0.78 ± 0.02	0.77 ± 0.02	0.75 ± 0.02 **

**p < 0.01, ***p < 0.001 vs. T0. Data are reported as the mean ± SEM. T1: 5–7 Weeks; T2: 11–21 Weeks.
